# Non- or minimally displaced distal radius fractures in adult patients < 50 years of age: Three weeks of cast immobilisation versus one week of brace immobilisation: study protocol for a multicentre randomised controlled trial

**DOI:** 10.1186/s13063-024-08375-4

**Published:** 2024-08-16

**Authors:** Eva A. K. van Delft, Suus G. J. van Bruggen, Nico L. Sosef, Frank W. Bloemers, Niels W. L. Schep, Jefrey Vermeulen

**Affiliations:** 1 Department of Trauma Surgery, Amsterdam UMC, Meibergdreef 9, Amsterdam, 1105 AZ The Netherlands; 2 Department of Surgery, Spaarne Gasthuis Hospital, Boerhaavelaan 22, Haarlem, 2035 RC The Netherlands; 3 Department of Trauma and Hand Surgery, Maasstad Hospital, Maasstadweg 21, Rotterdam, 3079 DZ The Netherlands

**Keywords:** Distal radial fractures, Wrist fractures, Non-operative treatment, Conservative treatment, Immobilisation period

## Abstract

**Background:**

Currently, non- or minimally displaced distal radius fractures are treated by 3 to 5 weeks of cast immobilisation. Many patients with a distal radius fracture suffer from long-term functional restrictions, which might be related to stiffness due to cast immobilisation. Current literature indicates that 1 week of immobilisation might be safe; however, no level 1 evidence is available. This trial aims to compare 1 week of brace immobilisation with 3 weeks of cast immobilisation in patients with distal radius fractures that do not need reduction.

**Methods:**

The aim of this trial is to evaluate the non-inferiority of 1 week of brace immobilisation in patients with non- or minimally displaced distal radius fractures. A two-armed single blinded multicentre randomised clinical trial will be conducted in three hospitals. Adult patients, between 18 and 50 years old, independent for activities of daily living, with a non- or minimally displaced distal radius fracture can be included in this study. The intervention group is treated with 1 week of brace immobilisation, and the control group with 3 weeks of cast immobilisation. Primary outcome is the patient-reported outcome measured by the Patient-Related Wrist Evaluation score (PRWE) at 6 months. Secondary outcomes are patient-reported outcome measured by the Quick Disabilities of the Arm, Shoulder and Hand score at 6 weeks and 6 months, PRWE at 6 weeks, range of motion, patient-reported pain score measured by VAS score, radiological outcome (dorsal/volar tilt, radial height, ulnar variance, presence of intra-articular step off), complications and cost-effectiveness measured by the EuroQol 5 Dimension questionnaire, Medical Consumption Questionnaire and Productivity Cost Questionnaire.

**Discussion:**

This study will provide evidence on the optimal period of immobilisation in non-operatively treated displaced and reduced distal radius fractures. Both treatment options are accepted treatment protocols and both treatment options have a low risk of complications. Follow-up will be according to the current treatment protocol. This study will provide level 1 evidence on the optimal period and way of immobilisation for non- or minimally displaced distal radius fractures in adult patients.

**Trial registration:**

ABR 81638 | NL81638.029.22 | www.toetsingonline.nl. 18th of October 2023

**Supplementary Information:**

The online version contains supplementary material available at 10.1186/s13063-024-08375-4.

## Background

The prevalence of distal radius fractures (DRFs) is bimodal; there is a peak at childhood age, concerning mostly male patients, followed by a peak in patients above 70 years of age, mostly woman [1]. Approximately 10–35% of all distal radius fractures (DRFs) are non- or minimally displaced (radial shortening < 3 mm, dorsal tilt < 10°, volar tilt < 20°) [2, 3]. Currently, the preferred treatment for non- or minimally displaced DRFs is immobilisation for 3 to 5 weeks in a cast or brace [4].

Literature shows that wrist joint stiffness is correlated with a longer period of immobilisation [5, 6]. Wrist stiffness may lead to a restricted range of motion and worse functional outcome. It is believed that stiffness is caused by restricted movement during cast immobilisation over a longer period of time [5]. The ideal treatment of DRF will be short, safe and will lead to an early return to work or daily activities, which is especially important in young and vital patients. In the past, non- or minimally displaced DRFs were immobilised for 4 to 6 weeks [7, 8]. Literature suggests that a shorter period of immobilisation is safe and might even accelerate and enhance functional outcome [9–12]. Recently, a systematic review on the duration of cast immobilisation concluded that a maximum of 3 weeks of cast immobilisation is equal in functional outcome and will not lead to more complications compared with a longer period of immobilisation in both displaced and non-displaced DRFs. Results of this study were limited by strength of available evidence as well as bias. Also, data were too heterogeneous to be pooled to perform a meta-analysis [13]. Beside functional benefits, it is plausible that shortening the period of immobilisation will aid independency and accelerated return to work or school. However, some fear complications such as secondary displacement and pain [9]. Secondary displacement is described as a complication of the non-operatively treated DRF, which occurs in 90% of the cases in the first 2 weeks following trauma, predominantly in displaced and closely reduced DRFs [14, 15]. Secondary displacement in non- and minimally displaced DRFs is very uncommon [3, 10, 16]. Furthermore, two studies stated that the only advantage of cast immobilisation within this group is the antalgic function, which might also support shorter treatment in vital patients [10, 17]. Bracing may provide a comfortable alternative to cast immobilisation. It will allow earlier movement and return to work and might lead to higher patient satisfaction and improvement of cost-effectiveness. Bracing is not related to increased risk of secondary displacement or other complications, as the fractures analysed in this study are non- or minimally displaced and therefore stable fractures. Many studies have been published on functional bracing in DRFs, none providing level 1 evidence [18–20].

This study was initiated to assess whether a period of 1 week of brace immobilisation within non- or minimally displaced DRFs is safe and will lead to an equal functional outcome, earlier return to work, higher patient satisfaction and reduction of health care costs compared to 3 weeks of cast immobilisation. The hypothesis of the study is that 1 week of brace immobilisation is non-inferior to 3 weeks of cast immobilisation.

This randomised controlled trial will provide level 1 evidence on the optimal period and way of immobilisation for non- or minimally displaced distal radius fractures in adult patients. Literature indicates that a shorter period of immobilisation and immobilisation by bracing is safe and might even be beneficial.

This study protocol is based on the ‘DR PIP II: Distal Radius Plaster Immobilization Period II’ study protocol (NL62861.029.17) where 4 weeks versus 6 weeks of cast immobilisation is analysed within displaced and reduced DRFs [21].

## Methods/design

The aim of this trial is to evaluate the non-inferiority of 1 week of brace immobilisation in patients with non- or minimally displaced distal radius fractures. As the ideal treatment of DRF is short, safe and will lead to an early return to work or daily activities, non-inferiority of 1 week of brace immobilisation will be considered superior to a longer period of immobilisation. This hypothesis was also supported by the previous study performed by this study group [22].

Primary outcome is:

- Patient-reported outcome measured by the PRWE score after 6 months

Secondary outcomes:

- Patient-reported outcome measured by the Quick Disabilities of the Arm, Shoulder and Hand score (qDASH) after 6 weeks and 6 months
- Patient-reported outcome measured by the PRWE score after 6 weeks
- Range of motion after 6 weeks and 6 months
- Patient-reported pain score (VAS score) at arrival at ED, after cast/brace is applied, in the first week after brace/cast removal, at 6 weeks and 6 months
- Radiological outcome at 6 weeks and 6 months
- Complications
- Cost-effectiveness measured by EQ-5D, iMCQ and iPCQ questionnaires

The PRWE is a validated 15-item (scored 1–10) self-reported questionnaire and is the best questionnaire for evaluating the patient-reported outcome in case of DRFs [23, 24]. Patients rate their outcome on a 0–10 scale on pain and functional outcome, and scores will be transformed to a 0–100 score. A higher score indicates greater disability. The DASH Outcome Measure is a validated 30-item self-reported questionnaire for patients with disorders of the arm, shoulder and hand [25, 26]. The QuickDASH (qDASH) is the shortened version of the DASH Outcome Measure, using 11 items instead of 30 (scored 1–5) to measure pain and functional outcome. At least 10 of the 11 items must be completed to calculate a score. The scores will be transformed to a 0–100. A higher score indicates greater disability.

Range of motion will be measured by using a goniometer: volar/dorsal flexion, radial/ulnar deviation and pronation/supination will be determined. Patient-reported pain score will be measured by using the VAS score. VAS score is a widely used method for pain assessments. Patients score their pain on a scale of 1–10. A higher score indicated a higher level of pain. The VAS score will be measured on the ED at arrival and after application of the brace of cast. Patients will also receive a dairy after cast removal to record their pain score.

Radiological outcome will be measured by the principal investigators of the participating hospitals; data will be cross checked by the clinical investigator; dorsal and volar tilt in degrees, radial height in millimetres and ulnar variance in millimetres will be calculated directly after trauma and after 6 weeks and 6 months. Complications contain secondary displacement (radial shortening > 3 mm, dorsal tilt > 10° or intra-articular step-off > 2 mm), delayed/non-union, re-interventions, complex regional pain syndrome (CRPS), tendon injuries (ruptures and tendinitis), nerve injuries (carpal tunnel syndrome and lesions) and distal radial ulnar joint disability.

Cost-effectiveness and cost-utility of non- or minimally displaced DRFs treated by 3 weeks of cast immobilisation or 1 week of brace immobilisation will be analysed. The perspective of this study is societal, and therefore both direct and indirect costs due to the DRF will be considered. Direct costs will include treatment, follow-up visits at the outpatient clinic, additional visits to health care professionals and the treatment of complications. Non-health care related costs include expenses to travel to and from the hospital. Indirect costs are based on absenteeism of work or loss of working hours due to cast-immobilisation or pain. To estimate health care costs, the friction-cost method will be used to analyse the loss of production for an individual worker. This analysis is based on a cost-effectiveness analysis of a study on the non-operative versus the operative treatment of DRFs [27]. To analyse cost-effectiveness and cost-utility, the EuroQol 5D (EQ-5D), Productivity Cost Questionnaire (iPCQ) and the Medical Consumption questionnaires (iMCQ) will be used [28–31]. Only the applicable items are included in this study. To create baseline data, patients will be asked to complete those questionnaires at the ED at the day of trauma (*t* = 0). Patients will also be asked to complete these questionnaires at 6 weeks and 6 months.

**Fig. 1:**
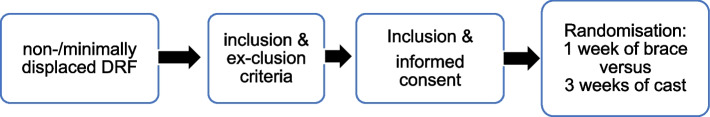
Inclusion procedure at ED

**Fig. 2:**
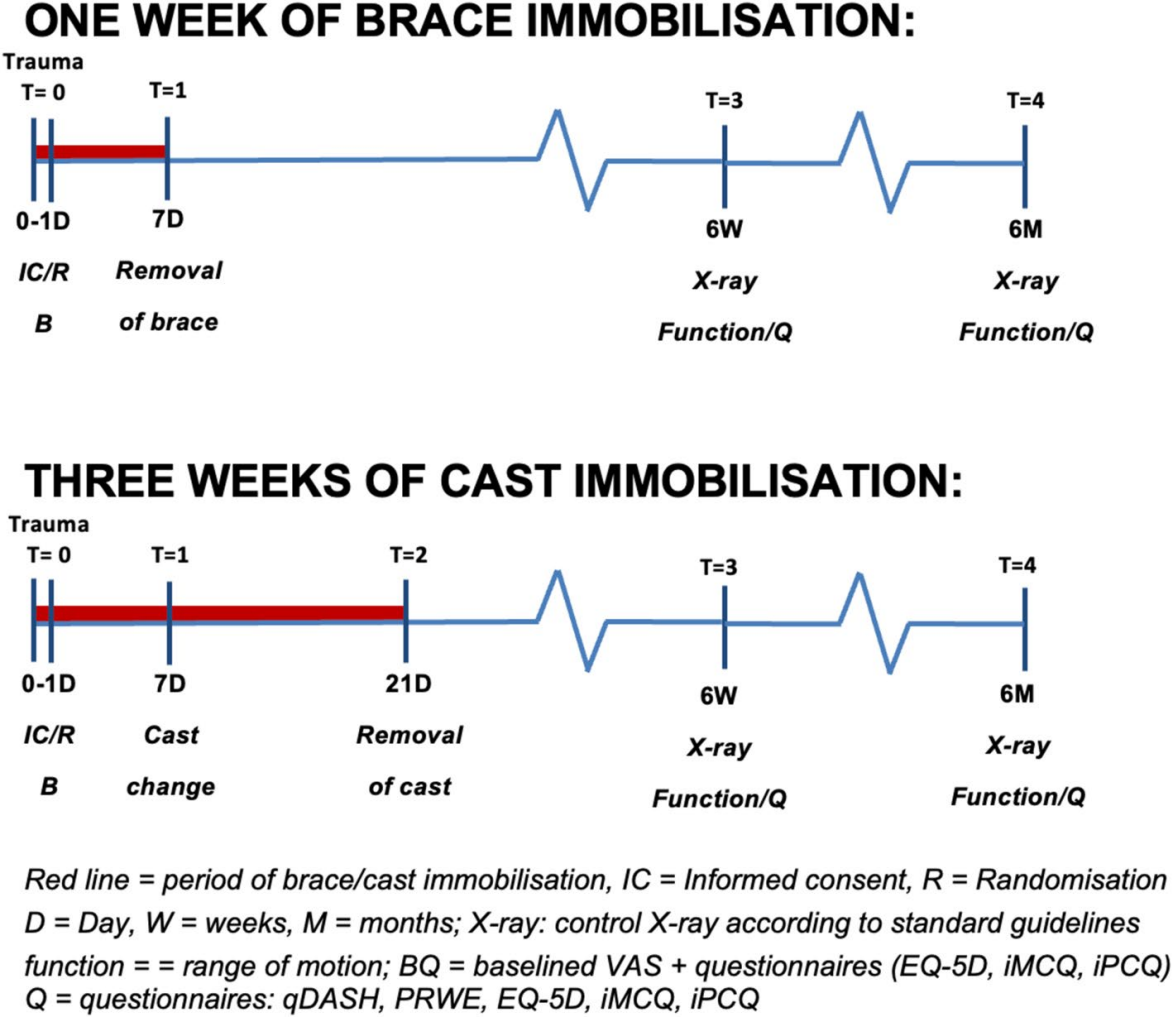
Follow-up scheme

This study will be conducted as a single-blinded multicentre randomised clinical trial in three hospitals. In this study, 3 weeks of cast immobilisation is compared to 1 week of brace immobilisation. The inclusion procedure and the moment of randomisation are visualised in Figs. 1 and 2. Patients will be treated in a lower arm cast in a neutral position or in a prefabricated brace [32]. The follow-up after the immobilisation period will be the same for both groups and will be according to the current treatment protocol. Additional physiotherapy is advised and exercises to train wrist function will be given. The follow-up is 6 months. We used the SPIRIT checklist when writing our report [33].

### Sample and selection criteria

The study population is defined as adult patients, between 18 and 50 years old, with a non- or minimally displaced DRF. Patients above the age of 50 are excluded from this study to eliminate bias by osteoporosis.

Inclusion criteria are:

- 18–50 years (to eliminate osteoporosis)
- Primary non- or minimally displaced DRF
- Independent for activities of daily living

Exclusion criteria are:

- Fracture of the contralateral wrist
- Ipsilateral fractures, proximal of the DRF
- Pre-existent abnormalities or functional deficits of the fractured wrist
- Open fractures
- Language disability to understand the Dutch patient information and questionnaires

Patients with a non- or minimally displaced DRF will be initially managed on the emergency department (ED) by X-ray and cast immobilisation in a lower arm cast in a neutral position [32]. Non- or minimally displaced will be classified according to the guidelines of the American Association of Orthopedic Surgeons (AAOS) and the Dutch guidelines. They state that the following radiographic parameters do not need a reduction before immobilisation: radial shortening <3 mm, dorsal tilt <10°, volar tilt <20° or intra-articular step-off <2 mm, >15° radial inclination, intact radiocarpal alignment and absence of radial or dorsal/volar translation [4, 34].

Evaluation of eligible patients will take place either at the ED or at the outpatient department of Spaarne Gasthuis, Haarlem/Hoofddorp, Amsterdam UMC, location AMC, Amsterdam and Maasstad Hospital, Rotterdam, the Netherlands. Patients with a DRF will be treated by the physician on call at the ED. Patients are eligible if they meet the in- and exclusion criteria. If a patient is eligible, he/she will start with the standard treatment for non- or minimally displaced DRF, namely cast immobilisation.

Patients can only participate if the DRF is non- or minimally displaced. They will receive written information and an informed consent form from the attending physician/the clinical investigator/research assistant. When the X-ray taken at the ED showed non- or minimal displacement, informed consent will be signed and eligible patients will be randomised (Fig. 1). An independent research assistant will perform a concealed permuted block randomisation by using a secured computer-generated randomisation schedule after stratification for gender and age. Allocation will be at random in four blocks (Table 1). In total, 1 week of brace immobilisation, if assigned to the intervention group, or 3 weeks of cast immobilisation, if assigned to the control group, will be executed. It is expected that all patients can be included within 1–1.5 year from the start of the study.

**Table 1:** Stratification by gender and age

Patient characteristics			Randomisation
List 1	Male	< 30 y.o.a	ABAB AABB ABBA BABA BAAB
List 2	Male	> 30 y.o.a	BAAB BBAA ABAB AABB ABBA
List 3	Female	< 30 y.o.a	AABB ABBA BAAB BBAA BABA
List 4	Female	> 30 y.o.a	ABBA BABA ABAB AABB AABB

*y.o.a.* years of age, *A* 1 week of brace immobilisation, *B* 3 weeks of cast immobilisation

The inclusion and follow-up schedule are visualised in Fig. 2 and Table 2. All patients will be followed for 6 months in total. Assessments will occur at the time of admission at the ED, assessment by phone consult in 1 week after trauma (5–9-day window) in case of inclusion in the intervention group (e.g. 1 week of brace immobilisation), physical assessment in case of the control group (e.g. 3 weeks of cast immobilisation) at 1 (5–9-day window) and 3 weeks (18–24-day window). All patients will be physically assessed at 6 weeks (5–7-week window) and 6 months (5–7-month window) after the start of treatment. After removal of the brace or cast, additional physiotherapy is advised and exercises to train wrist function will be given.

**Table 2:**
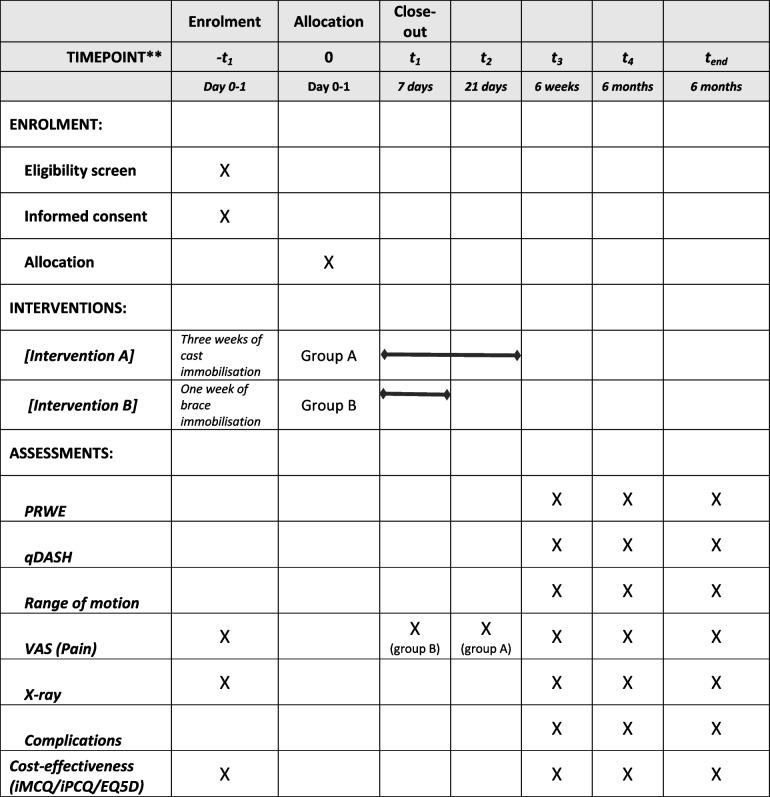
SPIRIT figure

At each follow-up (FU) visit, the research coordinator or research assistant will ascertain patient status (i.e. secondary interventions, adverse events/complications, deaths) and will verify the information within medical records. All adverse events will be addressed to the principal investigator. The patients will be asked to indicate the actual pain level based on the VAS score, if they are currently treated by a physical therapist and if they returned to work. At 6 weeks and 6 months, the range of motion of the wrist will be measured using a goniometer.

Patients will be asked to complete questionnaires relating to disability (qDASH score and PRWE score) and medical consumption, productivity and general health to analyse cost-effectiveness (EQ-5D, iPCQ, iMCQ). Patients will be asked to complete the questionnaires at the ED as well, to generate baseline data.

Plain X-rays of the wrist will be made at the time of presentation in the hospital at the ED, after 6 weeks and 6 months. The X-ray at 6 months will be taken to determine the grade of degenerative joint changes. Time to define the presence of a delayed- or malunion will be at 6 months. Intention-to-treat principle will be maintained.

Both treatment options and used methods of treatment for this type of fracture are well known. Earlier research did not report any (serious) adverse events. All adverse events will be reported by the investigator; severe adverse events will be reported by the investigator to the sponsor of the study without undue delay after obtaining knowledge of the events. We performed an investigator-initiated risk classification: the risk of this study is expected to be negligible. Therefore, no DSMB/safety committee would be necessary. A monitoring audit will be conducted at the start of the study, after inclusion of the first 10 patients and after finishing the inclusion of patients. Also, a line listing of (serious) adverse events will be kept up and reported annually to the Amsterdam UMC Ethical Board.

### Sample size calculation

The primary outcome will be the PRWE score at 6-month follow-up, of which the minimal clinically important difference is 11.5 points. To be conservative, we use a non-inferiority limit of 10.0. The SD of the PRWE is 14.0 [35]. With a power (1 − *β*) of 90% and a type I error (*α*) of 5%, a sample size of 34 patients per treatment group was calculated.

To allow a 10% drop-out, 74 patients will be included, 37 patients per treatment arm. We expect that all patients can be included within 1–1.5 year.

### Statistical analysis

Data from the demographic data collection and the outcome parameters will be cleaned blindly from the treatment data. Data are presented as mean scores with 95% confidence intervals. Linear regression analysis will be performed if data are not normally distributed.

A blinded evaluation of the trial patient’s functional status will be performed by a research assistant by use of the PRWE and qDASH scores. Blinded radiographic evaluation will be performed the intervention group and the control group. An independent expert will assess the degree of dislocation on an X-ray, blinded from the first assessment. Final blinded analysis of the primary outcome parameters will only be performed after 6-month follow-up.

The analysis of this study will be carried out according to the intention-to-treat principle, i.e. the patients will remain in the group they will be randomly allocated to at the baseline. Analysis of functional outcome will be assessed using repeated-measures analysis of variance (GLM 4) with the time as the within-group factor and the treatment as the between-group factor. The post hoc analysis will be performed on the time of randomisation. Group comparisons at different time points will only be made when the overall repeated-measures tests are statistically significant. All scores will be tested for normality using the Kolmogorov–Smirnov test. Parametric variables will be compared using the Student’s *t*-test, while non-parametric and ordinal variables will be compared using the Mann–Whitney *U* statistic. Nominal variables will be compared across independent groups using the chi-squared test or Fisher’s exact test. Homogeneity of variance will be assessed using Levene’s test.

Baseline characteristics that will be analyses are gender, age, dominancy, fracture side, fracture characteristics, smoking and osteoporosis. The outcome will be tested for normality and according to the outcome mean or median baseline characteristics will be compared.

Primary and secondary outcome measures are functional outcome (PRWE and qDASH), return to work, range of motion, pain, radiological outcome, complications and cost-effectiveness.

Missing data of the PRWE and qDASH score during follow-up can be imputed using multiple imputation based on baseline characteristics

The PRWE questionnaire will measure the primary outcome (PRWE after 6-month follow-up). In this questionnaire, pain and function are scored on a 0 to 10 scale. The total score can range between 0 and 100, with 0 being the best possible outcome and 100 the worst. The outcome will be tested for normality, and according to the outcome mean or median PRWE after 6 months will be calculated. The mean or median outcome after 6 months in the intervention group (e.g. 1 week of brace immobilisation) will be compared with the control group (e.g. 3 weeks of cast immobilisation). A *t*-test will be used to compare the different outcomes. A *p* value of < 0.05 will be taken as a threshold of statistical significance. To check for clinically relevant results, we will compare the outcomes between the two groups to check whether this difference will reach the minimal clinically important difference of the PRWE of 11.5. Different variables will be compared between the two treatment groups (e.g. 1 week of brace and 3 weeks of cast immobilisation groups). In addition, if possible, a multivariate analysis will be performed, to compare different secondary outcomes to the period of brace or cast immobilisation. Depending on the amount of missing data, we will impute outcomes using multiple imputation based on age, sex, smoking and fracture characteristics. For all analysis, SPSS statistical software (version 28.0) will be used, in which two-tailed *p* value < 0.05 will be considered significant.

### Ethical approval

This study had been approved by the Amsterdam UMC Ethical Board. Written, informed consent to participate will be obtained from all participants.

### Clinical trial registration

The study protocol of this study has been published on toetsingonline.com, trial registration of the Dutch Central Committee on Research Involving Human Subjects that meets the requirements of the ICMJE: https://www.toetsingonline.nl/to/ccmo_search.nsf/fABRpop?readform&unids=CEE3EDCF24F7B5B4C1258A64000B484F.

## Discussion

Three weeks immobilisation for non- or minimally displaced DRF is proven to be safe and sufficient [12].

In this study, adults < 50 years of age with a non- or minimally displaced DRF will be randomised between 3 weeks of cast immobilisation and immobilisation by a brace for 1 week. This option of treatment has been described in literature and guidelines, but is not generally used [18, 20, 34].

The incidence of volar plating as treatment of DRFs has been increasing, especially in displaced DRFs [36]. Non- or minimally displaced DRFs do not need reduction and operative fixation and have been treated by cast immobilisation since history [37].

Literature shows that wrist joint stiffness is correlated with a longer period of immobilisation [5, 6]. Wrist stiffness may lead to a restricted range of motion and worse functional outcome. It is believed that stiffness is caused by restricted movement during cast immobilisation over a longer period of time [5]. The ideal treatment of DRF will be as short as possible, safe and will lead to an early return to work or daily activities, which is especially important in young and vital patients. Still, level I evidence on this subject is rare. Both the Cochrane Review and the AAOS do not provide a clear treatment advice on the optimal period of immobilisation and do not favour immobilisation by cast or by brace [32, 38].

Therefore, it is important to provide level I evidence on bracing of DRFs. This study is designed as a randomised controlled trial with a non-inferiority design. Also, cost-effectiveness will be taken into account. The shorter the period of immobilisation, the earlier independency and return to work or school can be achieved, which will improve cost-effectiveness as well. Primary outcome of this study is the PRWE score after 6-month follow-up. The sample size of this study is based on the MCID of the PRWE. Functional self-reported outcome is considered as most important outcome factor. X-rays will also be analysed; however, secondary dislocation is not expected, as the non-displaced DRFs included in this study are considered to be stable. Furthermore, the correlation between radiological outcome and patient-reported outcome has been disputed in literature before. Functional outcome, especially patient-reported outcome, is the most important outcome factor. Radiological outcome does not always correlate to functional outcome, especially when aberrant outcome is subtle. But as very poor radiological outcome might influence other outcome factors, we chose to analyse this factor as well as a secondary outcome [39, 40].

This study will provide evidence on the optimal period of immobilisation in non-operatively treated displaced and reduced distal radius fractures. Both treatment options are accepted treatment protocols and both treatment options have a low risk of complications. Follow-up will be according to the current treatment protocol. This study will provide level 1 evidence on the optimal period and way of immobilisation for non- or minimally displaced distal radius fractures in adult patients.

## Trial status

Protocol number: 5, October 2023.

Date start of recruitment: approx. December 2024.

Approximate date recruitment to be completed: approx. December 2025.

## Supplementary Information

Additional file 1. SPIRIT checklist

## Data Availability

Data available upon request.

## Abbreviations

DRF: Distal radius fracture
qDASH: Quick Disabilities of the Arm, Shoulder and Hand score
PRWE: Patient-Related Wrist Evaluation
VAS: Visual analogue scale
ED: Emergency department
EQ-5D: EuroQol Group
iMCQ: I Medical Consumption Questionnaire
iPCQ: I Productivity Cost Questionnaire
AAOS: American Association of Orthopedic Surgeons
FU: Follow-up
X-ray: Radiography
GLM: Generalised linear regression
MCID: Minimum clinically important difference
